# Investigation of retinal microvasculature and choriocapillaris in adolescent myopic patients with astigmatism undergoing orthokeratology

**DOI:** 10.1186/s12886-022-02572-y

**Published:** 2022-09-23

**Authors:** Xiao-qin Wang, Ming Chen, Liu-zhi Zeng, Long-qian Liu

**Affiliations:** 1 Department of Ophthalmology, West China Hospital, Sichuan University, No. 37, Guoxue Lane, Chengdu, Sichuan Province 610041 China; 2 The Department of Ophthalmology, Chengdu First People’s Hospital/Chengdu Integrated TCM & Western Medicine Hospital, Chengdu, Sichuan Province 610041 China

**Keywords:** Myopia, Astigmatism, Orthokeratology, Optical coherence tomography angiography, Retinal thickness, Choroid, Microcirculation

## Abstract

**Background:**

To observe alterations of fundus microcirculation and retinal thickness in adolescent myopic patients with astigmatism after toric and spherical orthokeratology using optical coherence tomography angiography (OCTA), to explore the effects of orthokeratology on the retinal thickness and choroidal blood flow.

**Methods:**

A total of 48 patients were enrolled and divided into two group (toric orthokeratology (T) group and spherical orthokeratology (S) group) according to the type of lens design. OCTA was used to measure the superficial and deep retinal vessel densities at the macular region, radial peripapillary capillary (RPC) density, foveal avascular zone (FAZ) area, and choriocapillaris (ChC) perfusion area before and after orthokeratology for 3 months. The data were statistically analyzed by SPSS 19.0 software.

**Results:**

Compared with before orthokeratology, the superficial vessel density in the fovea and parafovea in the T group significantly increased, and the deep vessel density in the whole area and fovea were significantly elevated after 3 months (*P* < 0.05). The superficial vessel density was significantly higher only in the parafovea in the S group after 3 months than that before orthokeratology (*P* < 0.05), deep vessel density in the whole area and parafovea after 3 months was significantly higher than that before orthokeratology (*P* < 0.05). RPC density in the two groups increased after 3 months of orthokeratology in the whole area and inside the disc area (*P* < 0.05).

Three months after toric orthokeratology, FAZ area in the T group was significantly reduced by 0.05 (− 0.41 to + 0.08) mm^2^, while ChC perfusion area was enlarged by 0.06 ± 0.12 mm^2^. FAZ area in the S group significantly decreased by 0.01 (− 0.19 to + 0.01) mm^2^, whereas ChC perfusion area increased by 0.06 (− 0.07 to + 0.50) mm^2^. Retinal thickness in the two groups increased after 3 months of orthokeratology in the whole area and parafoveal area (*P* < 0.05).

**Conclusion:**

Orthokeratology improved retinal blood flow in macular area and RPC while controlling myopia. The changes in FAZ and ChC perfusion areas did not significantly differ between toric and spherical orthokeratology.

## Background

At present, myopia is the most common refractive error worldwide, influencing 80% of Asian adolescents [1]. If it is not effectively treated in time, it may lead to blinding complications, such as myopic macular disease, choroidal neovascularization, retinal detachment, cataract and glaucoma, causing irreversible visual impairment [2]. Orthokeratology has been proved to be an effective optical intervention in reducing axial length (AL) elongation and myopic progression [3]. Optical coherence tomography angiography (OCTA) has emerged as a noninvasive and quantitative imaging technique that allows stratified evaluation of the retinal and choroidal vasculature [4, 5]. OCTA shows three-dimensional maps of the macular and papillary vessel network, and it assesses the vessel density in different layers without any requirement for contrast agents [6]. As the OCTA can be performed in a short period of time without any side effects, it is straightforward to perform OCTA even in children [7]. Previous studies have reported high levels of repeatability and reproducibility of OCTA in the measurement of microvascular density of the macula and optic nerve head [8, 9].

Chen et al. found that choroidal thickness (ChT) of myopic children increased after short-term orthokeratology, which could be attributable to the altered retinal defocus profile associated with orthokeratology [10]. Then, whether choroidal and retinal blood flow will also be changed in myopia patients wearing orthokeratology lens. The relationship between the changes of fundus microcirculation and myopia control in adolescent myopia with orthokeratology lens is unclear. To date, macular and peripapillary vessel densities in myopia after short-term orthokeratology have not been investigated. Therefore, the present study aimed to observe the alterations of the fundus microcirculation and the retinal thickness in adolescent myopic patients with astigmatism after toric and spherical orthokeratology using OCTA, to explore the effects of orthokeratology on the retinal and choroidal blood flow, and to further analyze the mechanism of orthokeratology in the control of myopia.

## Methods

### Study subjects

In this cross-sectional study, 48 Chinese children were recruited from Contact Lens Clinic and Myopia Clinic of Chengdu First People’s Hospital between January 2020 and December 2020. The inclusion criteria for subjects were as follows: (1) age between 8 and 15 years, (2) myopic refractive error of − 5.00 to − 0.75 D, (3) with-the-rule astigmatism from 0.50 D to 2.00 D of axes 180 ± 20°, (4) best-corrected visual acuity (BCVA) equal to or better than 0.0 logMAR, (5) wearing orthokeratology lenses at least 8 h per night, (6) intraocular pressure (IOP) of 10–21 mmHg, (7) binocular anisometropia < 1.00 D, flat keratometry (K_f_): 39.00–46.00 D and steep keratometry (K_S_): 40.00–48.00 D, (8) Endothelial cell density (ECD) > 2500 cells/mm^2^. The exclusion criteria were as follows: (1) being allergic to orthokeratology or care solution, (2) patients with a history of ocular diseases and undergoing intraocular surgery, (3) patients with a history of receiving medicinal treatment that might affect vision or vision development.

According to the corneal curvature and corneal elevation difference, toric and spherical orthokeratology lenses were selected, which were divided into toric orthokeratology lens (T) group and spherical orthokeratology lens (S) group. Subjects in the T group were fitted with toric lens whose eyes had corneal astigmatism more than 1.50 D or corneal elevation difference between horizontal axis and vertical axis at 8-mm chord is more than 30 μm showed in corneal topographic height map. Subjects in the S group were fitted with spherical lens. If both eyes had the same astigmatism, the data of the right eye was analyzed. For the toric and spherical orthokeratology lenses in the current study, participants were fitted with four-zone reverse-geometry lenses (Boston XO material by Oupukangshi, Hefei, China). The oxygen permeability is 100 DK Units (gas to gas method). The overall diameters is 10.4 - 10.8 mm and the back optic zone diameter is 6.0 mm. The reverse curve radius is 0.4–1.0 mm and the center thickness is 0.24 mm. Appropriate lenses were initially fitted and fluorescein staining pattern was used to observe the fit of the lens in eye under a slit lamp. All subjects were instructed to return for a follow-up visit after 1 day, 1 week and one, three, six, and 12 months. At each visit, the participants underwent a slit-lamp examination to check for the lens related complications and any adverse events. Ocular biometrics and OCTA were measured at baseline. At 3-month visit OCTA was measured again.

Sample size was calculated based on the mean and standard deviation (SD) of ChT in a previous study, which investigated the effect of orthokeratology on ChT [10]. A sample size of at least 18 was required. Ophthalmologic examinations included BCVA, slit-lamp and fundoscopy. IOP was measured by non-contact tonometer (TX-10 tonometer, Canon, Japan). ECD was examined by corneal endothelial microscope (SUOER SW-7000, China). Schirmer I test was performed to assess the tear secretion without previous instillation of topical anesthetic for 5 min. Standardized strips special filter paper strip of 35 length and 5 mm width were placed in the lateral third of the lower eyelid, and the length of the moistened portion of the strip was observed. Myopic refractive error was obtained by cycloplegic refraction (RKT-700A; NIDEK, Gamagori, Japan). AL and white to white (WTW) were measured by IOL-Master system (IOL Master, Zeiss, Germany). Five continuous measurements were conducted, and the average data were collected for analysis. Corneal topography measurements were performed with the Placido ring-based Medmont topographer (SUOER SW-6000, China). K_f,_ K_S,_ corneal astigmatism and corneal elevation difference were obtained from the topography map with optimal quality.

### OCTA examination and scanning protocols

**Fig. 1:**
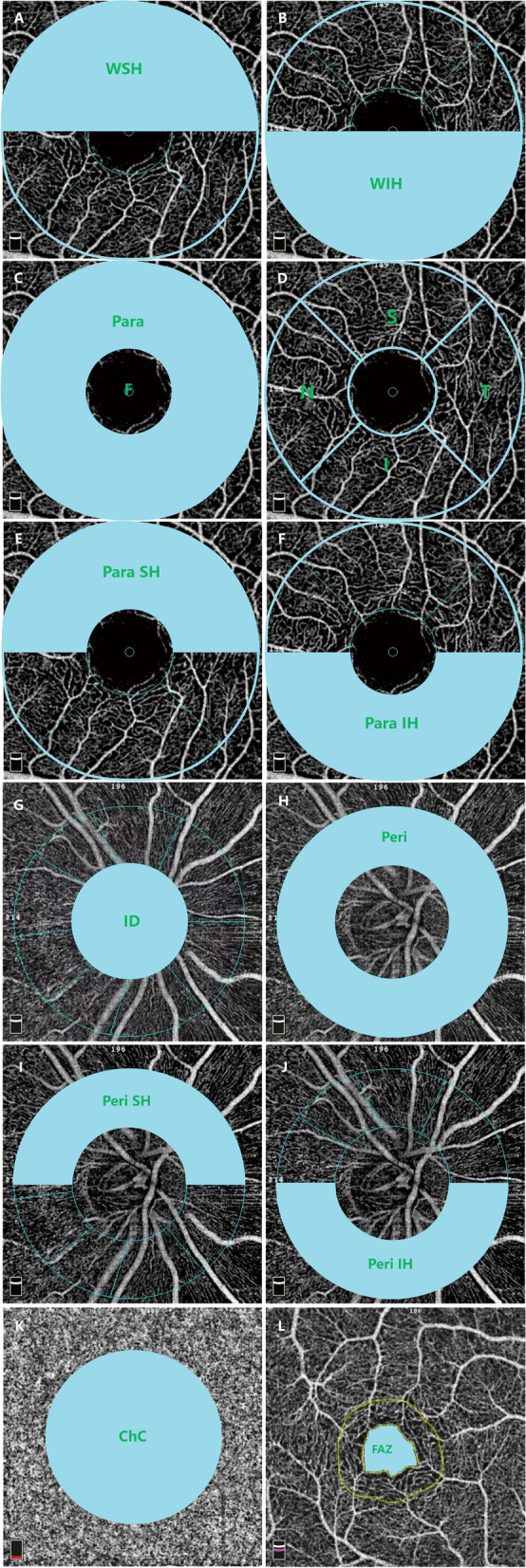
OCTA map. **A**: Whole Superior-Hemi, WSH; **B**: Whole Inferior-Hemi, WIH; **C**: Fovea, F and Parafovea, Para; **D**: Superior, S, Inferior, I, Nasal, N, Temporal, T; **E**: Superior-Hemi, Para SH; **F**: Inferior-Hemi, Para IH; **G**: Inside Disc, ID; **H**: Peripapillary, Peri; **I**: Superior-Hemi, Peri SH; **J**: Inferior-Hemi, Peri IH; **K**: Choriocapillaris flow area centered on the fovea, ChC; **L**: Foveal avascular zone, FAZ

OCTA was carried out using RTVue XR Avanti (Optovue Inc. Fremont, CA, USA) with the HD Angio Retina of 3.0 mm and the HD Angio Disc of 4.5 mm. The OCTA instrument software’s automatic segmentation was used to generate images of superficial capillary plexus (SCP), deep capillary plexus (DCP), and choriocapillaris (ChC). The SCP layer was defined from the inner limiting membrane to 10 μm above the inner plexiform layer, and the DCP layer was extended from 10 μm above the inner plexiform layer to 10 μm below the outer plexiform layer. Macular flow density was separately calculated based on the Early Treatment Diabetic Retinopathy Study (ETDRS) contour. The whole image of macular area was the average blood flow density of the whole circle with a diameter of 3 mm and divided in 2 hemispheres (Whole Superior-Hemi, WSH) (Fig. 1A) and (Whole Inferior-Hemi, WIH)(Fig. 1B). Fovea (F) referred to the blood flow density in a ring of 1.0 mm diameter, and parafoveal area (Para), as defined by the 3 mm partial ETDRS grid from the AngioVue software, was the area comprised between the 1–3 mm concentric ring centered of the fovea (Fig. 1C). The parafoveal area was then further divided into 4 quadrant sections, including superior (S), inferior (I), nasal (N), and temporal (T) sections (Fig. 1D). The parafoveal area was also divided into 2 hemispheres (Para Superior-Hemi, Para SH) and (Para Inferior-Hemi, Para IH), that were distinguished by a horizontal line through the foveal center (Fig. 1E,F). In addition, the macular thickness was automatically calculated in micrometer.

The software provided the whole en face flow density, inside disc (ID) flow density, peripapillary (Peri) and its Superior-Hemi (Peri SH), Inferior-Hemi (Peri IH) flow density (Fig. 1G-J). Moreover, the peripapillary retinal nerve fiber layer (pRNFL) thickness was also quantified in the radial peripapillary capillary (RPC) segment.

The ChC layer was automatically segmented from 10 μm above the Bruch’s membrane (BM) to 30 μm below the BM. ChC density was calculated as vascular areas of ChC divided by selected areas of 3.144 mm^2^ centered on the fovea (Fig. 1K) [11]. Subfoveal choroidal thickness (SFChT) was defined from the retinal pigment epithelium to the posterior margin of scleral junction, and it could be quantified by the OCT instrument. The FAZ area (mm^2^) was defined as the avascular area in the center of the fovea, which could be evaluated by the non-flow area tool to provide automated FAZ segmentation (Fig. 1L).

The image quality index was assessed automatically by the OCTA instrument, ranging from 1 to 10. OCTA scans were repeated until high-quality images were obtained. OCTA images were excluded if the image quality index was less than 7 and motion artifacts were found. All OCTA examinations were carried out by a single experienced examiner between 14:00 PM and 17:00 PM, and the quality of the images was individually assessed by two researchers (WXQ and MC).

### Statistical analysis

The statistical analysis was performed using the SPSS 19.0 software (IBM, Armonk, NY, USA). Data were presented as mean ± standard deviation or median (range). The normality of variables was assessed using the Shapiro-Wilk test. *P* < 0.05 was considered statistically significant. The paired t-test or Wilcoxon signed-rank test was used to compare differences between two groups or compare to baseline. The correlation between the retinal vessel density and retinal thickness at baseline was assessed using the Pearson or Spearman correlation analysis for both groups.

## Results

### Patients’ demographic characteristics

A total of 48 patients were enrolled in the present study, including 24 patients in the T group (male (12) vs. female (12)) who aged 8–15 years old, with an average age of 11.08 ± 2.04 years old. Besides, there were 24 patients in the S group (male (11) vs. female (13)) who aged 8–15 years old, with an average age of 11.29 ± 2.05 years old. Cylindrical refractive error, steep-corneal curvature and Schirmer test in the T group were slightly higher than those in the S group (*P* < 0.05), and there was no significant difference in other parameters between the two groups before orthokeratology (*P* > 0.05, Table 1).

**Table 1:** Participants’ characteristics at baseline

	T group	S group	*P*
Age (yr)	11.08 ± 2.04	11.29 ± 2.05	0.726^a^
Sphere (D)	- 3.00 ± 1.20	- 1.88(− 5.00 ~ − 0.75)	0.055^b^
Cylinder (D)	−1.13 (−2.00 ~ − 0.75)	- 0.50(− 1.00 ~ − 0.50)	**< 0.001**^**b**^
AL (mm)	24.85 ± 1.01	24.54 ± 0.71	0.229^a^
K_f_ (D)	42.93 ± 1.16	42.63 ± 1.36	0.414^a^
K_s_ (D)	44.72 ± 1.22	43.55 ± 1.43	**0.004**^**a**^
WTW (mm)	12.35 (11.60 ~ 12.80)	12.03 ± 0.35	0.063^b^
IOP (mmHg)	16.29 ± 2.40	17.08 ± 2.28	0.248^a^
Schirmer test (mm)	21.75 ± 2.36	19.46 ± 3.50	**0.011**^**a**^
ECD (cells/mm^2^)	2970.86 ± 165.15	2875.58 ± 175.53	0.059^a^

Significant *P* values are in bold

Values are shown as mean ± standard deviation and median (range)

^a^Paired t-test

^b^Wilcoxon signed-rank test

*AL* axial length, *K*_*f*_ flat keratometry, *K*_*s*_ steep keratometry, *WTW* white to white, *IOP* intraocular pressure, *ECD* Endothelial cell density

### Vessel density, FAZ and ChC in two groups

The macular SCP vessel density in the fovea and parafovea in the T group significantly increased after 3 months. The macular SCP vessel density only in the parafovea in the S group after 3 months was significantly higher than that pre-orthokeratology (*P* < 0.05, Table 2). The macular DCP vessel density in the whole area and fovea in the T group significantly increased after 3 months. The macular DCP vessel density in the whole area and parafoveal area in the S group after 3 months was significantly higher than that pre-orthokeratology (*P <* 0.05, Table 2). The RPC vessel density in both two groups increased after 3 months of orthokeratology in the whole area and inside disc area (*P* < 0.05, Table 2).

**Table 2:** Vessel density, FAZ and ChC before and after orthokeratology in two group

	T group		*P*	S group		*P*
	Baseline	three months		Baseline	three months	
SCP vessel density (%)						
Whole area	48.7 ± 2.1	49.2 ± 2.6	0.459^a^	48.8 ± 2.3	49.7 ± 2.4	0.171^a^
Superior-Hemi	48.5 ± 1.9	49.0 ± 2.9	0.551^a^	48.8 ± 2.0	49.8 ± 2.2	0.121^a^
Inferior-Hemi	49.0 (40.9–52.7)	49.3 ± 2.2	0.616^b^	48.8 ± 2.8	49.8 ± 2.5	0.220^a^
Fovea	19.7 (14.2–30.2)	23.3 ± 5.0	**0.002**^**b**^	19.8 ± 5.3	21.6 ± 5.4	0.103^a^
Parafovea	50.6 ± 2.3	52.7 ± 2.7	**0.003**^**a**^	51.2 ± 2.5	53.3 ± 2.1	**0.004**^**a**^
Superior-Hemi	51.4 ± 1.9	51.6 ± 3.6	0.864^a^	51.6 ± 2.3	52.5 ± 2.6	0.210^a^
Inferior-Hemi	51.3 ± 2.5	52.2 ± 2.8	0.240^a^	51.9 ± 3.0	53.3 ± 2.5	0.102^a^
Tempo	50.5 (41.8–54.9)	50.6 ± 3.3	0.658^b^	50.6 ± 2.9	51.9 ± 2.4	0.126^a^
Superior	52.5 ± 2.3	52.9 ± 3.5	0.655^a^	52.4 ± 2.9	54.0 ± 2.7	0.098^a^
Nasal	50.7 ± 2.3	51.2 (39.1 ~ 55.7)	0.943^b^	51.0 ± 2.4	51.8 ± 2.9	0.302^a^
Inferior	51.8 (41.7–57.1)	53.3 ± 3.6	0.189^b^	53.0 (40.8–57.1)	54.2 ± 2.7	0.089^b^
DCP vessel density (%)						
Whole area	53.5 ± 3.4	55.7 ± 2.6	**< 0.001**^**a**^	52.8 ± 3.0	54.8 ± 2.8	**0.001**^**a**^
Superior-Hemi	54.7 ± 3.4	55.3 ± 3.3	0.464^a^	53.5 ± 3.1	54.9 ± 3.4	0.052^a^
Inferior-Hemi	54.8 (45.2–59.2)	54.5 ± 2.6	0.573^b^	52.7 ± 3.1	54.0 ± 2.9	0.052^a^
Fovea	36.3 ± 4.5	39.3 ± 6.5	**0.013**^**a**^	34.2 ± 5.6	35.3 ± 5.1	0.340^a^
Parafovea	56.2 ± 3.2	57.3 ± 3.2	0.113^a^	55.0 ± 2.7	57.0 ± 2.9	**0.001**^**a**^
Superior-Hemi	56.7 ± 3.1	57.2 ± 3.8	0.465^a^	55.7 ± 2.8	57.1 ± 3.5	**0.044**^**a**^
Inferior-Hemi	56.0 ± 3.7	57.0 ± 2.9	0.217^a^	54.9 ± 2.8	56.4 ± 2.9	**0.019**^**a**^
Tempo	56.7 ± 3.1	57.3 ± 2.7	0.367^a^	55.8 ± 2.6	57.0 ± 2.8	**0.045**^**a**^
Superior	56.9 ± 3.5	57.7 ± 3.6	0.319^a^	55.6 ± 3.0	57.1 ± 3.9	0.119^a^
Nasal	56.6 ± 2.8	57.5 ± 3.0	0.088^a^	55.8 ± 2.8	56.8 ± 3.7	0.101^a^
Inferior	55.4 (45.5–61.2)	56.2 ± 3.9	0.297^b^	54.2 ± 3.3	55.9 ± 3.0	0.027^a^
RPC vessel density (%)						
Whole area	49.6 ± 2.1	51.3 (49.1–56.3)	**0.001**^**b**^	49.3 ± 2.2	50.9 ± 2.1	0.005^a^
Inside disc	50.9 ± 5.5	56.5 (39.1–61.8)	**< 0.001**^**b**^	51.3 ± 4.6	55.2 ± 3.7	**0.003**^**a**^
Peripapillary	52.0 ± 2.5	52.7 ± 2.4	0.367^a^	51.9 ± 2.3	52.3 ± 2.1	0.478^a^
Superior-Hemi	52.3 ± 2.6	53.0 ± 2.1	0.344^a^	52.3 ± 2.2	52.6 (42.4–57.7)	0.568^b^
Inferior-Hemi	51.8 ± 2.9	53.0 ± 3.1	0.224^a^	51.9 ± 2.7	52.1 ± 2.6	0.811^a^
FAZ (mm^2^)	0.27 ± 0.06	0.24 ± 0.07	**0.024**^**a**^	0.31 ± 0.07	0.27 ± 0.07	**0.002**^**a**^
ChC (mm^2^)	2.18 ± 0.10	2.26 ± 0.10	**0.004**^**a**^	2.19 ± 0.08	2.27 (2.11–2.80)	**< 0.001**^**b**^

Significant *P* values are in bold

Values are shown as mean ± standard deviation and median (range)

^a^Paired t-test

^b^Wilcoxon signed-rank test

*FAZ* foveal avascular zone, *ChC* choriocapillaris, *SCP* superficial capillary plexus, *DCP* deep capillary plexus, *RPC* radial peripapillary capillary

Three months after toric orthokeratology, FAZ area in the T group significantly decreased by 0.05 (− 0.41 to + 0.08) mm^2^, while ChC perfusion area was enlarged by 0.06 ± 0.12 mm^2^. FAZ area in the S group was significantly reduced by 0.01 (− 0.19 to + 0.01) mm^2^, whereas ChC perfusion area was enlarged by 0.06 (− 0.07 to + 0.50) mm^2^. There were no significant differences in the changes of FAZ and ChC perfusion areas between the two groups (*P* > 0.05) (Table 2).

### Vessel density, FAZ, ChC and SFChT of different ages in two groups

For subjects of age from 8 to 11 in T group after wearing orthokeratology 3 months, macular SCP vessel density in the fovea significantly increased (*P* < 0.05, Table 3). For subjects of age from 11 to 15 after wearing toric orthokeratology 3 months, macular SCP and DCP vessel density in the whole area and parafovea significantly increased, and ChC perfusion area was enlarged (*P* < 0.05, Table 3). In both two age subgroups, RPC vessel density increased in the inside disc area and SFChT was elevated after 3 months of toric orthokeratology (*P* < 0.05, Table 3).

**Table 3:** Vessel density, FAZ, ChC and SFChT of two age groups before and after toric orthokeratology

	8-11y (*n* = 12)		*P*	12-15y (*n* = 12)		*P*
	Baseline	three months		Baseline	three months	
SCP vessel density(%)						
Whole area	48.6 ± 2.4	49.0 ± 2.5	0.679^a^	48.8 ± 1.9	49.3 ± 2.8	**0.027**^**a**^
Fovea	19.8 (16.0–28.3)	23.5 ± 4.4	**0.034**^**b**^	19.7 ± 4.4	23.1 ± 5.8	0.060^a^
Parafovea	50.6 ± 2.4	52.2 ± 2.9	0.147^a^	50.7 ± 2.4	53.1 ± 2.6	**0.003**^**a**^
DCP vessel density (%)						
Whole area	52.1 ± 3.0	55.2 ± 2.4	0.005^a^	54.9 ± 3.3	56.1 ± 2.9	**0.010**^**a**^
Fovea	37.9 ± 5.4	40.2 ± 7.1	0.145^a^	34.7 ± 2.9	38.4 ± 6.2	0.054^a^
Parafovea	55.4 ± 2.9	56.3 ± 3.0	0.507^a^	57.0 ± 3.5	58.4 ± 3.2	**0.016**^**a**^
RPC vessel density(%)						
Whole area	49.1 ± 1.6	51.7 (50.5–56.3)	**0.005**^**b**^	50.1 ± 2.4	51.4 ± 2.2	0.075^a^
Inside disc	52.0 ± 4.9	56.0 ± 3.7	**0.007**^**a**^	49.8 ± 6.0	53.4 ± 6.2	**0.002**^**a**^
FAZ (mm^2^)	0.25 ± 0.07	0.24 ± 0.07	0.397^a^	0.29 ± 0.06	0.20 (0.16–0.37)	0.050^b^
ChC (mm^2^)	2.24 (2.02–2.27)	2.18 ± 0.09	0.084^b^	2.19 ± 0.11	2.28 ± 0.11	**0.024**^**a**^
SFChT (μm)	282.7 ± 23.2	298.4 ± 21.0	**0.000**^**a**^	291.3 ± 30.3	304.6 ± 26.9	**0.000**^**a**^

Significant *P* values are in bold

Values are shown as mean ± standard deviation and median (range)

^a^Paired t-test

^b^Wilcoxon signed-rank test

*FAZ* foveal avascular zone, *ChC* choriocapillaris, *SFChT* subfoveal choroidal thickness, *SCP* superficial capillary plexus, *DCP* deep capillary plexus, *RPC* radial peripapillary capillary

For subjects of age from 8 to 11 after wearing spherical orthokeratology 3 months, macular DCP vessel density in the whole area significantly increased (*P* < 0.05, Table 4). For subjects of age from 11 to 15 after wearing spherical orthokeratology 3 months, macular SCP vessel density in the whole area, fovea and parafovea significantly increased, and DCP vessel density increased in the whole area and parafovea (*P* < 0.05, Table 4). Besides, RPC vessel density increased in the whole area and inside disc (*P* < 0.05, Table 4). In both two age subgroups after 3 months of spherical orthokeratology, ChC perfusion area was enlarged, SFChT was elevated, and FAZ area significantly decreased (*P* < 0.05, Table 4).

**Table 4:** Vessel density, FAZ, ChC and SFChT of two age groups before and after spherical orthokeratology

	8-11y(*n* = 11)		*P*	12-15y(*n* = 13)		*P*
	Baseline	three months		Baseline	three months	
SCP vessel density(%)						
Whole area	49.3 ± 2.1	49.0 ± 2.3	0.792^a^	48.4 ± 2.5	50.4 ± 2.3	**0.026**^**a**^
Fovea	21.2 ± 5.3	21.4 ± 6.5	0.951^a^	18.6 ± 5.2	21.8 ± 4.4	**0.002**^**a**^
Parafovea	51.7 ± 2.6	52.6 ± 1.9	0.448^a^	50.8 ± 2.4	53.9 ± 2.2	**0.000**^**a**^
DCP vessel density(%)						
Whole area	51.6 (0.3–57.0)	54.3 ± 3.2	**0.026**^**b**^	53.3 ± 3.7	55.1 ± 2.5	**0.019**^**a**^
Fovea	36.4 ± 6.6	36.4 ± 5.7	0.978^a^	32.4 ± 4.1	34.3 ± 4.6	0.060^a^
Parafovea	54.2 ± 1.9	6.1 ± 2.7	0.074^a^	55.6 ± 3.2	57.7 ± 3.0	**0.005**^**a**^
RPC vessel density(%)						
Whole area	49.4 ± 2.1	50.8 ± 1.9	0.088^a^	49.2 ± 2.3	51.0 ± 2.3	**0.032**^**a**^
Inside disc	52.6 ± 5.5	55.9 ± 2.5	0.151^a^	50.3 ± 5.0	54.5 ± 4.5	**0.006**^**a**^
FAZ (mm^2^)	0.29 ± 0.08	0.27 ± 0.07	**0.037**^**a**^	0.33 ± 0.06	0.27 ± 0.07	**0.014**^**a**^
ChC (mm^2^)	2.17 ± 0.07	2.24 ± 0.06	**0.030**^**a**^	2.25 (2.06–2.30)	2.31 (2.11–2.80)	**0.011**^**b**^
SFChT (μm)	274.5 ± 30.1	287.5 ± 26.1	**0.001**^**a**^	272.8 ± 28.7	288.9 ± 30.6	**0.001**^**a**^

Significant *P* values are in bold

Values are shown as mean ± standard deviation and median (range)

^a^Paired t-test

^b^Wilcoxon signed-rank test

*FAZ* foveal avascular zone, *ChC* choriocapillaris, *SFChT* subfoveal choroidal thickness, *SCP* superficial capillary plexus, *DCP* deep capillary plexus, *RPC* radial peripapillary capillary

### Thicknesses of macular, choroid, and RNFL before and after orthokeratology in two group

Retinal thickness in the two groups increased after 3 months of orthokeratology in the whole area and parafoveal area (*P* < 0.05). Besides, SFChT was elevated by 14.0 ± 6.3 and 14.7 ± 9.0 μm in the T and S groups, respectively. There was no significant difference in pRNFL thickness before and after orthokeratology for 3 months (*P* > 0.05). The pRNFL thickness in the T group was129.5(108 - 150) mm before orthokeratology and 123.5(108 - 156) after orthokeratology for 3 months. The pRNFL thickness in the S group decreased after 3 months of orthokeratology (*P* < 0.05) (Table 5).

**Table 5:** Thicknesses (μm) of macular, choroid, and RNFL before and after orthokeratology in two group

	T group		*P*	S group		*P*
	Baseline	three months		Baseline	three months	
Whole area	309.7 ± 11.7	314.9 ± 10.9	**0.040**^**a**^	313.6 ± 15.1	319.9 ± 16.1	**0.004**^a^
Superior-Hemi	310.5 ± 12.2	316.0 ± 11.2	0.053^a^	313.3 ± 15.1	319.6 ± 15.3	**0.004**^a^
Inferior-Hemi	308.5 ± 11.7	313.8 (281.0–327.0)	**0.004**^**b**^	314.8 ± 15.7	317.6 ± 16.0	0.276^a^
Fovea	250.5 ± 14.9	251.2 ± 12.9	0.807^a^	247.6 ± 16.8	245.8 ± 12.5	0.757^a^
Parafovea	317.8 ± 11.7	325.0 ± 12.2	**0.004**^**a**^	323.1 ± 15.6	329.2 ± 15.4	**0.004**^a^
Superior-Hemi	320.2 ± 13.3	325.6 ± 12.3	0.054^a^	325.6 ± 16.3	329.1 ± 16.3	0.110^a^
Inferior-Hemi	317.6 ± 11.8	323.9 ± 11.6	**0.010**^**a**^	323.3 ± 15.1	327.1 ± 16.5	0.097^a^
Temporal	309.7 ± 13.5	315.6 ± 12.2	**0.035**^**a**^	316.0 ± 14.4	320.0 ± 16.2	0.096^a^
Superior	322.4 ± 13.7	328.5 ± 12.7	**0.042**^**a**^	328.2 ± 17.1	332.8 ± 16.6	0.052^a^
Nasal	324.5 ± 12.0	327.6 ± 11.7	0.213^a^	329.2 ± 16.6	331.3 ± 16.9	0.329^a^
Inferior	318.6 ± 11.8	323.8 ± 12.2	**0.032**^**a**^	324.3 ± 15.6	328.3 ± 17.0	0.110^a^
SFChT	287.0 ± 26.8	301.5 ± 23.8	**< 0.001**^**a**^	273.6 ± 28.7	288.3 ± 28.0	**< 0.001**^a^
pRNFL	129.5 (108–150)	123.5 (108–156)	0.166^b^	127.0 (106–181)	126.1 ± 12.3	**0.038**^**b**^

Significant *P* values are in bold

Values are shown as mean ± standard deviation and median (range)

^a^Paired t-test

^b^Wilcoxon signed-rank test

*RNFL* retinal nerve fiber layer, *SFChT* subfoveal choroidal thickness, *pRNFL* peripapillary retinal nerve fiber layer

### Correlation among retinal thickness, AL, and retinal vessel density

Before orthokeratology, the AL in the T group was positively correlated with the SCP vessel density in the whole area (*r* = 0.429, *P* = 0.036) and foveal area (*r* = 0.422，*P* = 0.040). The SCP vessel density in the T group was positively correlated with the retinal thickness in the whole area (*r* = 0.476, *P* = 0.019) and foveal area (*r* = 0.519, *P* = 0.009). The DCP vessel density in the S group was negatively correlated with the retinal thickness in the whole area (*r* = − 0.433, *P* = 0.035) and foveal area (*r* = − 0.454, *P* = 0.026).

## Discussion

The retinal capillary network is divided into superficial layer and deep layer. The superficial layer of capillary network is mainly located in the nerve fiber layer and ganglion cell layer, while the deep layer of capillary network is mainly located in the core layer and outer plexus layer [12]. Fan et al. found that both SCP and DCP vessel densities were associated with AL and spherical equivalent. The density of retinal vessels decreased in macular region with the increase of myopia [13]. Another study demonstrated that the density of superficial retinal vessels decreased and FAZ area was enlarged in myopic children [14]. In the present study, it was found that the SCP vessel density increased in foveal and parafoveal areas in adolescent myopic patients after 3 months of the toric orthokeratology, while the DCP vessel density was elevated only in the parafoveal area of adolescent myopic patients after 3 months of the spherical orthokeratology. The changes in SCP and DCP vessel densities mainly occurred in the foveal area after the treatment of myopic patients by toric orthokeratology, while those changes were mainly observed in parafovea area after spherical orthokeratology.

The ChC is a layer of capillary with the thickness of about 10 μm, which is located at the innermost part of the choroid, between the BM and the middle choroidal vascular layer [15]. ChT and choroidal blood perfusion were significantly reduced in myopic guinea pig eyes, and changes in ChT were positively correlated with changes in choroidal blood perfusion [16]. Scleral hypoxia can cause the scleral extracellular matrix remodeling, leading to the formation of myopia. It is plausible that scleral hypoxia in experimental myopia could be caused by the reduction of ChT and choroidal blood perfusion [17]. Orthokeratology can increase the adjustment function of adolescent myopic patients and reduce the adjustment lag, thereby controlling myopia. In the present study, it was found that after wearing orthokeratology for 3 months, the ChC perfusion area in adolescent myopic patients was enlarged, indicating that orthokeratology not only controlled the progression of myopia, but also improved choroidal blood circulation.

Choroid is rich in a large number of blood vessels, and the changes in its structure and function play a certain role in the development of myopia [18]. ChT is correlated with refractive diopters to a certain extent, and the higher the myopia degree, the thinner the ChT [19]. Changes in ocular refractive state can lead to rapid changes in ChT. Hyperopic defocus caused choroidal thinning, while myopic defocus induced relative choroidal thickening in children [20]. Several studies have shown that the control of myopia by orthokeratology may be related to the changes in ChT, and the increase of ChT after orthokeratology may be associated with the changes of defocus state of myopia by orthokeratology [21, 22]. Li et al. found that SFChT increased in children after orthokeratology, and the ChT would return to the baseline level 1 month after terminating the orthokeratology [23]. The results of the present study showed that SFChT of adolescent myopic patients increased after treatment with toric and spherical orthokeratology for 3 months, and there was no significant difference in SFChT between the two types of orthokeratology. The choroidal thickening caused by orthokeratology may affect the propagation path of light and cause changes in choroidal blood flow, thereby controlling the development of myopia. Thus, SFChT can be used as a predictor of therapeutic efficacy.

FAZ is a capillary-free area inside the macular region, surrounded by interconnected capillary beds, which is the most sensitive area of vision [24, 25]. FAZ mainly reflects the microcirculation status in macular area [26]. FAZ size may be related to age, gender, AL, retinal thickness, macular blood flow, RNFL thickness, segmentation method, etc. [27]. Shahlaee et al. found that the FAZ area of superficial layer in normal adults was 0.27 ± 0.101 mm^2^, and that of deep layer was 0.34 ± 0.116 mm^2^, as measured by OCTA [28]. The FAZ area of high myopic eyes was larger than that of emmetropia [29, 30]. In the present study, the FAZ area of adolescent myopic patients was reduced at 3 months after orthokeratology compared with before treatment, and the density of micro-vessels in macular area increased. Therefore, orthokeratology could improve retinal blood flow in macular area, while it could control myopia.

Previous studies have shown that retinal thickness in macular area of myopic eyes is thinner than that of emmetropia due to the elongation of AL in myopic eyes [31]. However, the retina in the parafoveal and the peripheral macular areas is subjected to a greater tensile force of the sclera, thus, the retina in the para-foveal and the peripheral macular areas is more prone to thinning than that in the fovea [32]. In the present study, the retinal thickness increased in the whole area and its inferior-hemi, parafoveal and its inferior-hemi, temporal, inferior, superior quadrant at 3 months after toric orthokeratology. In addition, retinal thickness increased in the whole area and its superior-hemi, and in parafoveal area at 3 months after spherical orthokeratology. Therefore, the retinal thickness increased in the two groups in the whole area and parafoveal area.

Milani et al. reported that the superficial vessel density in macular area of adults with high myopia was positively correlated with the retinal thickness [33]. Wu et al. found that the thicknesses of retina and choroid in adults with high myopia were thinner, and there was a negative correlation between the thickness of outer retina and the density of deep retinal vessels, suggesting that the deep retinal vessels might have a compensatory effect on the hypoxic environment of high myopia [34]. The results of the present study revealed that the SCP vessel density was positively correlated with the retinal thickness of the whole macular area and the central macular area in adolescent myopic patients with astigmatism above 1.00 diopter, indicating that distribution of more blood vessels was associated with the greater retinal thickness. The DCP vessel density in adolescent myopic patients with astigmatism within 1.00 diopter was negatively correlated with the retinal thickness in the whole macular area and in the central area, indicating that the distribution of deep retinal microvascular network was associated with the thinning of retinal thickness. The results of the present study were consistent with Milani et al.’s [33] and Wu et al.’s findings [34]. It can be concluded that the SCP vessel density is higher and the retinal thickness is greater in both adolescent and adult myopic patients. Nevertheless, the DCP vessel density is higher and the retinal thickness is thinner.

It is noteworthy that RPC comprises a unique plexus of capillary beds within the RNFL, playing a key role in providing nutrition for human retinal ganglion cell axons [35]. Studies have shown that the RPC density in myopic patients decreased, while the vessel density in the subtemporal and superior temporal regions did not significantly change, so as to meet the metabolic requirements of nerve fibers in the bow-tie region [36]. In the present study, the RPC density in two groups increased after 3 months of orthokeratology in the whole area and inside the disc area. Thinning of pRNFL can lead to the reduced demand for retinal blood supply, attenuated metabolic demand, and ultimately decreased RPC density, with a significant reduction in pRNFL thickness in high myopic eyes [37]. The present study revealed that pRNFL thickness decreased after 3 months of spherical orthokeratology, which is inconsistent with the increase of RPC density. Further studies are therefore required to clarify the influences of orthokeratology on the pRNFL thickness.

## Conclusions

The increased retinal vessel density, the reduced FAZ area, and the enlarged ChC perfusion area in adolescent myopic patients with astigmatism were observed after 3 months of orthokeratology. The changes in FAZ and ChC perfusion areas did not significantly differ between toric and spherical orthokeratology. Orthokeratology improved retinal blood flow in macular area and RPC while controlling myopia.

## Data Availability

The datasets used and/or analysed during the current study are available from the corresponding author on reasonable request.
